# In Situ Observation of Deformation-Induced Spherical Grains in Semi-Solid State of C5191 Copper Alloy

**DOI:** 10.3390/ma13235496

**Published:** 2020-12-02

**Authors:** Miao Cao, Jucun Wang, Qi Zhang, Ke Huang

**Affiliations:** 1 School of Mechanical Engineering, Xi’an Jiaotong University, Xi’an 710049, China; ke.huang@mail.xjtu.edu.cn; 2 State Key Laboratory of Materials Processing and Die & Mould Technology, Huazhong University of Science and Technology, Wuhan 430074, China; 3 Department of Egineering, AECC South Industry Co., Ltd., Zhuzhou 412002, China; wjc7260@163.com

**Keywords:** semi-solid, spherical grains, strain-induced, recrystallization, in-situ microstructure, Zener pinning, heterogeneous nucleation

## Abstract

The formation mechanism of spherical grains during the strain-induced melt activation is investigated by in situ observation of the cold rotary swaged materials during heat treatment. The microstructure of the cold rotary swaged material changed from original dendritic structure to spherical grains after heating semi-solid state, whereas the as-received alloy without deformation exhibited non-spherical grains. These results show that static recrystallization, preferential melting of grain boundaries, and small grains cause the deformed grains to form the initial spherical grain structure during the temperature rising to semi-solid state; besides, the Zener pinning effect of second-phase particle and the heterogeneous nucleation of solidification also play negative roles in spherical grain growth up freely during the cooling process.

## 1. Introduction

The strain-induced melt activation (SIMA) process is widely used to prepare semi-solid materials for a variety of industrial alloys, such as aluminum and magnesium alloys [1]. SIMA utilizes plastic deformation energy of the deformed material to obtain spherical grains during heat treatment [2]. The classic theory ascribes the formation of spherical grains to the recrystallization process and liquid phase penetration [3,4,5]. Atkinson et al. [4] have revealed that, when the worked materials are reheated into the semi-solid state, recrystallization did occur and, as liquids were formed, they penetrated the recrystallized grain boundaries to form spheroids. Lin et al. [6] have concluded that the α-Mg grains in the pre-deformed materials become rounder and finer after the semisolid isothermal heat treatment because of the recrystallization mechanism. However, other opinions can also be found in the literature. For instance, Hassas-Irani et al. [7] and Yu et al. [8] deduced that the dendritic grains melted first, and ripening grains with a spherical shape were subsequently formed under the difference of interface energy. Vieira et al. [1] have proposed that a dispersion of spheroidal Al-α particles was first formed in an Al–Si alloy due to the transformation of low angle boundaries into high angle boundaries, and the liquid phases were liable to penetrate the grain boundaries during the heating process. Besides, a semi-solid metal product is formed in the semi-solid state and, subsequently, the temperature will gradually cool to room temperature. The solidification method has an important influence on the growth and formation of spherical grains during the cooling process. Therefore, the solidification process of solid-liquid slurry needs be studied. It thus appears that there is no unified mechanism of the formation of spherical grains and the role of solidification process is ignored.

In order to investigate the formation mechanism of spherical grains, the real-time microstructural evolution should be observed during heat treatment. Usually, the microstructural evolution is studied by observing the morphology of post-mortem quenched samples at different melting states [9]. However, the quenched samples may suffer from the high diffusion rate of the elements and exhibit phase transformation during quenching [9]. Hence, the observation of actual microstructural changes during heat treatment remains an extremely challenging task. Therefore, in situ techniques are required to assess the microstructural changes during heating and cooling processes to overcome the disadvantages of post-mortem quenched samples. In this paper, we observed and analyzed the real-time microstructural evolution of deformed materials with different rotary swaged strains ε (ε = (*d*_o_ − *d*)/*d*_o_, where *d*_o_ is original diameter of as-received metal bars and *d* is the diameter of rotary swaged metal bars), during heat treatment by employing high-temperature confocal laser scanning microscopy (HT-CLSM).

## 2. Materials and Methods

### 2.1. Materials

Extruded C5191 Copper alloy was used as the raw material. The C5191 tin copper presented samples were composed of 93.2% of Cu, 6.2% of Sn, 0.2% of Ni, 0.3% of P, and other elements in insignificant amounts [10]. The differential scanning calorimetry demonstrated that the semi-solid state temperature range of C5191 copper alloy is between 916 and 1042 °C.

### 2.2. HT-CLSM In-Situ Observation Experiment

**Figure 1:**
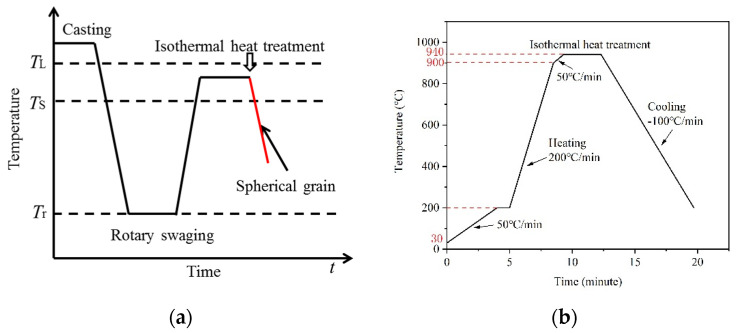
(**a**) Schematic diagram of the rotary swaging strain-induced melt activation (RSSIMA) process (*T*_L_: liquidus, *T*_S_: solidus, and *T*_r_: room temperature) and (**b**) actual heating cycle during high-temperature confocal laser scanning microscopy (HT-CLSM).

Herein, cold rotary swaging was utilized to deform the raw material, which exhibits columnar dendrites. Then, the deformed material was heated to the semi-solid state for several minutes, which leads to spherical grains [10]. The rotary swaging strain-induced melt activation (RSSIMA) process is schematically illustrated in Figure 1a. For comparison, the microstructural evolution of the as-received C5191 was studied under the same conditions, as presented in Figure 1b. A Lasertech VL2000DX-SVF17SP HT-CLSM (Lasertech, Yonekura, Japan) [11,12] was utilized to observe the in situ microstructural evolution of rotary-swaged (rotary swaged strain of 0.3) and as-received C5191 alloys (rotary swaged strain of 0) during heating/cooling cycles. During heating, the samples with the dimensions of Φ 7 mm × 3 mm were placed in an alumina crucible, and then positioned on a Pt stage, under an argon atmosphere to avoid oxidation.

### 2.3. Electron Backscattering Diffraction Analysis

The orientation distribution of rotary swaged C5191 and semi-solid C5191 was examined by electron backscattering diffraction (EBSD) analysis using the scanning electron microscope Zeiss Merlin (Zeiss, Oberkochen, Germany). The EBSD Kikuchi patterns were generated with an acceleration voltage of 20 kV and a data acquisition speed of 22 Hz. EBSD mappings were performed with a scan step of 0.7 μm.

## 3. Results

### 3.1. In Situ Microstructural Evolution of Deformed Alloy during the Heat Treatment Process

**Figure 2:**
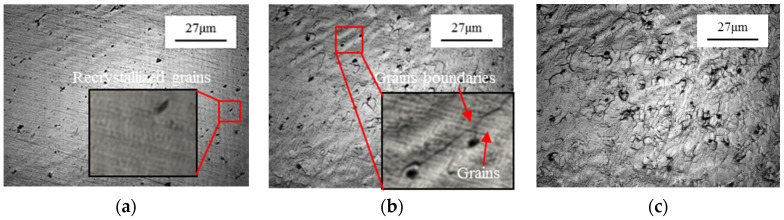

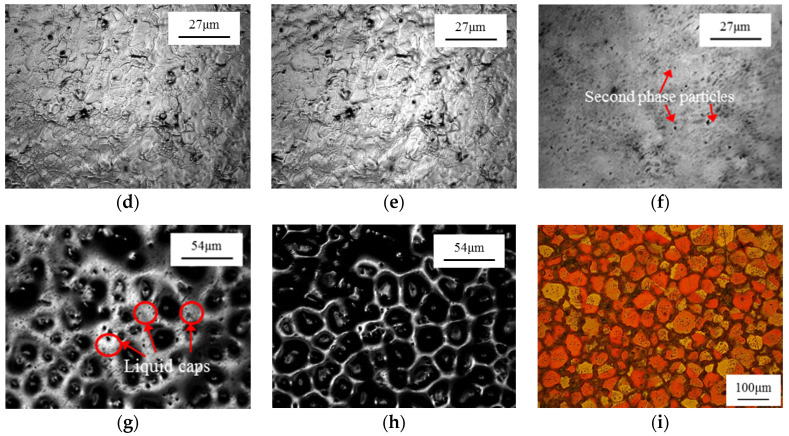
HT-CLSM-assisted in situ microstructural observations of rotary swaged C5191 alloy with rotary swaged strain of 0.3 during thermal cycle (Figure 1b) at different temperatures: (**a**) 606 °C, (**b**) 745.9 °C, (**c**) 852.0 °C, (**d**) 896.9 °C, (**e**) 897.8 °C, (**f**) 940 °C, (**g**) 902.5 °C, and (**h**) 892.2 °C; (**i**) optical image of solidified microstructure.

The in situ microstructural evolution of C5191 alloy, from the rotary swaged state to the semi-solid state, is presented in Figure 2. The specimen surface remained unchanged until 606 °C (Figure 2a), however, statically recrystallized grains [13] started to appear and the recrystallized grain exhibited a size (statically recrystallized grains size is average equivalent diameter $D=\sqrt{4A/\pi }$, where *A* is the area of the grain) of ~1–2 μm. Moreover, the freshly recrystallized grains started to replace the original deformed grains. At the same time, some black spots and grain boundary grooves emerged with increasing temperature (Figure 2b). At this stage, the recrystallized grains rapidly consumed the deformed grains through the grain boundaries migration as a result of the difference in stored energy in different grains. However, the migration of grain boundaries gradually slowed down because of the impingement of recrystallized grains and was further slowed down by the second-phase particles, as well as the formation of grain boundary grooves from the existing recrystallized grains. The size distribution of the grains is not uniform at the early stage. The mean size of recrystallized grains is about 1 μm, while the mean size of grains, induced by grain boundary grooves, is about 3 μm. At 896.9 °C, the recrystallized grain size was found to be ~4 μm. Moreover, the small grains, local points in the interior of grains and grain boundaries, are preferentially melted with increasing temperature (Figure 2e). The sample was held at the peak temperature of 940 °C for 4 min, followed by rapid cooling at 100 °C/min, which resulted in a large amount of precipitated second-phase particles in the microstructure (Figure 2f). In order to better observe the solidification process of the microstructure, the magnification of the microscope was adjusted, as shown in Figure 2g–h. During cooling, the precipitation of additional second-phase particles is observed at 902.5 °C (Figure 2g). It is well known that the liquid phase and solid phases coexist in the semi-solid mushy, which leads to the heterogeneous nucleation during the solidification process [14]. At 902.5 °C, liquid spherical caps (in the field of view of HT-CLSM, the lower position is black, and the raised position is bright) were formed on the sample surface because the second-phase particles could serve as inoculant during nucleation in solidification [15]. The liquid phase has substantially solidified at 892.2 °C. It is worth noting that a large amount of second-phase particles hindered liquid segregation and acted as a nucleation inoculant, inhibiting the growth of nucleated grains, which promoted the grain refinement [16]. Figure 2i presents that the rotary swaged dendritic microstructure has been successfully tailored to uniform spherical grains. The average equivalent diameter, *D* ($D=\sqrt{4A/\pi }$), and shape factor, *F*_S_ (*F*_S_ = 4π*A*/*P*^2^), of spherical grain were found to be 36.6 μm and 0.56, respectively.

### 3.2. In Situ Microstructural Evolution of As-Received Alloy during the Heat Treatment Process

**Figure 3:**
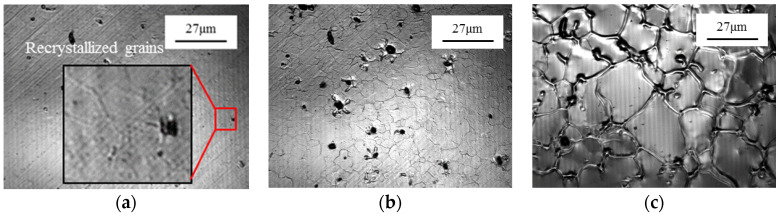

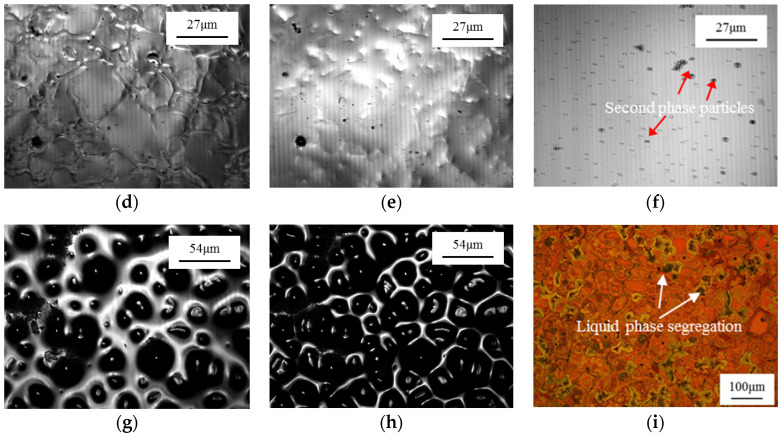
In situ HT-CLSM microstructural observations of as-received C5191 alloy during thermal cycle (Figure 1b) at different temperatures: (**a**) 645.7 °C, (**b**) 700.1 °C, (**c**) 890.1 °C, (**d**) 893.5 °C, (**e**) 895.9 °C, (**f**) 939.5 °C, (**g**) 878.9 °C, and (**h**) 873.5 °C; (**i**) optical image of solidified microstructure.

The in situ microstructural evolution of as-received C5191 alloy was observed from room temperature to the semi-solid state (Figure 3). Notably, no obvious change in microstructure was observed up to 645.7 °C (Figure 3a). At 700.1 °C, the statically recrystallized micro-grains emerged and covered the sample surface. The recrystallization starting temperature is clearly higher than its rotary swaged counterpart. The mean grain size was found to be ~6 μm and the grain boundaries became more evident. The recrystallized grains rapidly coarsened with increasing temperature because of the grain boundary migration (Figure 3b,c). Furthermore, the grain boundaries and local points in the interior of grains started to melt at 893.5 °C and the mean grain size increased to about 25 μm (Figure 3d). Moreover, the liquid phase diffused toward the grain interior and precipitated in the form of black spots at 895.9 °C (Figure 3e). When the temperature was further increased to 940 °C and held for 4 min, a large amount of second-phase particles appeared on the sample surface (Figure 3f). The solidification was carried out at a cooling rate of 100 °C/min and microstructural changes were recorded. Herein, heterogeneous nucleation occurred and the liquid phase was completely solidified at 878.9 °C (Figure 3g,h). During solidification, no obvious liquid caps appeared. The solidification microstructure of as-received C5191 alloy consists of coarse grains, a large number of rose-like grains, and a small number of spherical grains, wherein the maximum grain length was 500 μm (Figure 3i). Moreover, severe liquid phase segregation was observed in the microstructure.

These results reveal that only high-sized non-spherical grains can be formed in the raw material during semi-solid state temperature. Even though the heterogeneous nucleation during solidification can effectively increase the number of nucleation sites, and thus refine the grain size, the final solidification microstructure is not the fine-grained spherical structure. Therefore, the recrystallization effect alone is not sufficient to form fine spherical grains. In fact, the heterogeneous nucleation and Zener pinning effect of second-phase particles also played critical roles in the formation of near-spherical grains.

## 4. Discussion

### 4.1. Refinement Mechanism of Grains

**Figure 4:**
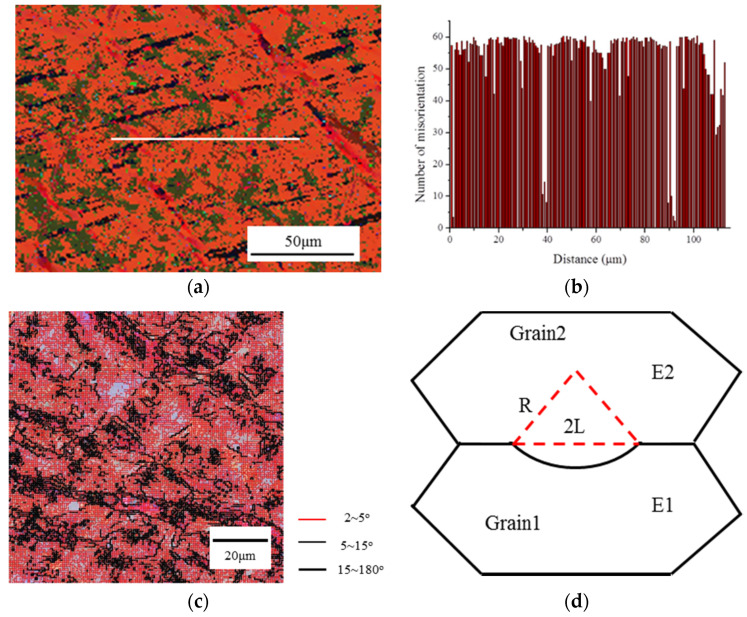
Rotary swaged microstructure with radial strain of 0.2 and semi-solid microstructure analyzed by electron backscatter diffraction (EBSD): (**a**) rotary swaged C5191 alloy orientation map [21], (**b**) misorientation distribution map corresponding to the line in (**a**) [21], (**c**) map of low-angle grain boundaries of rotary swaged C5191 alloy, and (**d**) strain induced grain boundary migration of a boundary separating a grain of low stored energy (E2) from one of higher energy (E1) adapted from [17].

The refinement mechanism of grains during strain-induced melt activation (SIMA) requires further explanation. Based on the static recrystallization theory, the recrystallization temperature increases with decreasing prior deformation strain and increasing starting grain size. Meanwhile, the recrystallized grain size highly depends on the deformation structure (e.g., deformation strain or dislocation densities), annealing conditions and orientations among neighboring grains, original grain size, solute and second-phase particles, and so on [17,18]. Figure 4 shows the misorientation distribution maps of the rotary swaged C5191 alloy with the radial strain of 0.2 by electron backscatter diffraction (EBSD). Orientations among neighboring grains are highly different in the line of the Figure 4a, due to both deformation banding and twinning [19], where a high number of boundaries exceeded 60°. Moreover, there is a high number of high-sized local misorientations and variations in the sub-grain morphology. Besides, the rotary swaged C5191 alloy has a high dislocations density due to the high proportion of low-angle grain boundaries (GBs) [20,21]. The increased driving force (stored energy) arising from the dislocations and particle stimulated nucleation, which are generated by the deformation, is well-known. Therefore, a higher deformation strain of the rotary swaged samples will provide more dislocations and a high amount of the misorientation of neighboring grains.

Once the stored energy is known, we can now turn to the critical size of static recrystallization [17]. The stored energy that drives the recrystallization process is very small. If two deformed grains have stored energies of *E*1 and *E*2 and *E*2 < *E*1, then the driving force is provided by the energy difference Δ*E* = *E*1 − *E*2, as shown in Figure 4d. If the bulging boundary is a critical size of nucleus radius *R_crit_*, with a specific boundary energy *E_GB_*, then the function can be expressed by the following [17]:

(1) $$R_{crit}>\frac{2E_{GB}}{\Delta E}$$

It is illustrated that the size of recrystallization grains decreases with the increase in the stored energy. Moreover, the rotary swaged material recrystallizes at a lower temperature than the raw material [18]. Therefore, both the higher stored energy and lower recrystallization temperature favor finer recrystallized grain size for the rotary swaged material.

Furthermore, the smaller recrystallized grain size provides more grain boundaries, which act as the precipitation sites for the second-phase particles. Zener pinning of the second-phase particle plays a major role in retarding primary recrystallization, which influences the grain coarsening. At semi-solid state temperature, the grain boundaries preferentially melted as a result of higher deformation energy. With finer grain structures, the interparticle spacing between the second-phase particles, decorated along the grain boundaries, is also decreased. These second-phase particles exhibited a prominent Zener pinning effect [17] and inhibited grain growth during semi-solid state soaking. Moreover, the growth of small-sized second-phase particles, which exist at fine grain boundaries, resulted in wetting of second-phase particles by the liquid phase. Moreover, some of these second-phase particles acted as nucleation sites for heterogeneous solidification, whereas others were pushed to the grain boundaries to hinder the primary grain growth during solidification by Zener pinning, resulting in the formation of spherical grains. Overall, the results reveal that a sufficiently large amount of deformation strain energy is required to form fine spherical grains.

### 4.2. Spheroidization Mechanism of Grains

The spheroidization mechanism of grains during SIMA needs to be further revealed. Assuming there is a solid particle in the liquid matrix and a solid–liquid interface, the interface energy must be overcome. The total Gibbs free energy of the tiny solid particles in the liquid matrix is inversely proportional to the size of the particles [22]. As the free enthalpy of the solid particle increases with the decrease of its diameter, while the free enthalpy of the liquid phase remains constant, the melting point of the solid particles in pure metal and alloy will decrease accordingly. The melting point drop caused by the radius of curvature *r* of the spherical grains in the melt can be written as follows [22]:

(2) $$\Delta T_{r}=\frac{2\Gamma }{r}$$

where Δ*T_r_* are curvature undercooling degree or Gibbs–Thomson undercooling degree/K, and Γ is the Gibbs–Thomson coefficient.

It can be seen that, the smaller the crystal grains, the more the melting point decreases from Formula (2). Therefore, the small crystal grains preferentially melt during the isothermal heat treatment (abbreviated as IHT) process. The melting mechanism schematic diagram of small grains and grain boundary intersections (sharp corner) during the heating process.

**Figure 5:**
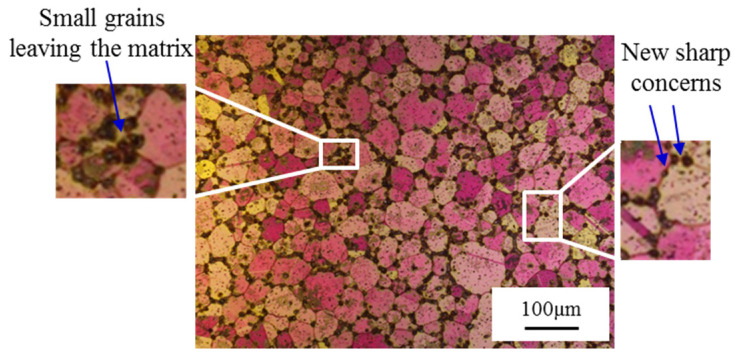
Microstructures of rotary swaged C5191 at the early stage of IHT.

The melting point of the grain boundary intersections drops with the increase in gains’ curvature. Moreover, small grains with a lower melting point are widely distributed in the grain boundaries and inside the grains. Therefore, for equiaxed crystals, the small crystal grains at sharp corners and solid particles on the grain boundary usually melt first. The microstructure of the early stage temperature holding period is shown in Figure 5. A discontinuous liquid phase appears on the grain boundary; at the same time, a large amount of small liquid phase is dispersed in the crystal grains. The liquid phase interface expands with the temperature rising or the holding time increasing. The sharp corners on the grain boundary will be separated from the matrix by the gradually connected liquid phase, forming small crystal grains, which have a lower melting point and melt. As time goes by, the grain boundaries of polygonal equiaxed crystals basically turn into arcs, forming a spherical shape with a liquid phase inside. Finally, a solid–liquid coexistence microstructure with spherical crystal grains is formed. In the same way, the equiaxed crystal structure in the raw material will also undergo a similar spheroidization process. It can be inferred that both the microstructures of the rotary swaged materials and raw materials may be spherical structures during the temperature rising and the holding period of the IHT. The spherical structure in Figure 2i and the remaining spherical structure in Figure 3i prove the correctness of the above theoretical inference.

The solidification process has an important influence on the final microstructure of the semi-solid alloys and semi-solid alloy products. Both microstructures of raw materials and rotary swaged materials are composed of solid grains and liquid matrix after the holding period in the semi-solid state, and heterogeneous nucleation solidification will occur during the cooling period of IHT.

**Figure 6:**
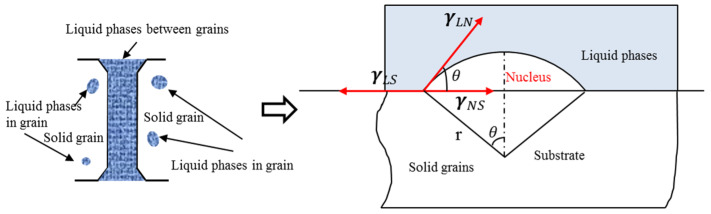
Principle of liquid phase heterogeneous nucleation.

The solid grains were considered as the substrate, whereas the residual solid alloy phases in the liquid phases were utilized as nuclei, which led to the generation of numerous new nuclei [22]. The schematic of heterogeneous nucleation in the mushy zone is presented in Figure 6. When the nucleus of new grains comes into contact with the substrate, a contact angle (wetting angle) θ and a sphere crown with critical size are formed. Assuming the nucleus is part of the sphere crown. The relationship between wetting angle θ and interfacial free energy can be expressed as follows [23]:

(3) $$\gamma_{\mathrm{LN}}\cos\theta +\gamma_{\mathrm{NS}}=\gamma_{\mathrm{LS}}$$

where γ_LN_, γ_NS_, and γ_LS_ are the liquid/nucleus, nucleus/substrate, and liquid/substrate interfacial energies, respectively.

The volume of the new grain can be represented as follows [23]:

(4) $$V=\int_{0}^{\theta }\pi {\left(r\sin\theta \right)}^{2}d\left(r-r\cos\theta \right)=\frac{{\pi r}^{3}}{3}\left(2-3\cos\theta +\cos^{3}\theta \right)$$

where V is a geometric shape determined by the wetting angle θ and (180° − θ) is the measured contact angle between the melt and the substrate.

The difference of free energy is derived from the energy balance relationship as follows [22]:

(5) $$\Delta G_{k}^{*}=\Delta G_{k}f\left(\theta \right)=\Delta G_{k}\left(\frac{\left(2+\cos\theta \right){\left(1-\cos\theta \right)}^{2}}{4}\right)$$

where $\Delta G_{k}^{*}$ is the difference of heterogeneous nucleation free energy and $\Delta G_{k}$ is the difference of homogeneous nucleation free energy.

The nucleation model is determined by the wetting angle θ [22]. When 0° < wetting angle θ < 180°, the nucleation model is heterogeneous nucleation. The closer the wetting angle is to 180°, the more spherical the crystal nucleus. When the wetting angle θ = 180°, the nucleation method is homogeneous nucleation. When θ = 0°, the grains grow up with no nucleation obstacle [22]. Comparing the microstructures of the melting process early stage of the rotary swaged material and the raw material, it is found that, when the temperature drops, the following occurs:

1. The liquid in rotary swaged material rapidly forms many spherical caps on the sample surface under the surface tension and Zener pinning effect of second-phase particles (Figure 2g).
2. The liquid in raw material remains flat under the surface tension (Figure 3g).

The wetting angles of the rotary swaged material are closer to 180°, while the wetting angle of the raw material is closer to 0°. Therefore, in the solidification process of the rotary swaged material, heterogeneous nucleation occurred and the grains were refined [24], and the final microstructure is a spherical structure (Figure 2i). Meanwhile, the raw material had no nucleation barriers during the solidification process, and the microstructure grows up directly and forms a thick rosette-like and long strip-like dendrite (Figure 3i).

### 4.3. Mechanism of Deformation-Induced Spherical Grains in Semi-Solid State

**Figure 7:**
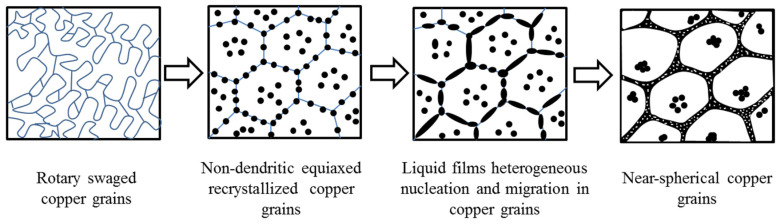
Schematic diagram of microstructure evolution of the rotary swaged alloys during RSSIMA.

These radiographic observations presented in Figure 2 and Figure 3 reveal that only high-sized dendrites can be formed in the raw material during semi-solid state temperature. Even though the heterogeneous nucleation during solidification can effectively increase the number of nucleation sites, and thus refine the grain size, the final solidification microstructure is not the fine-grained spherical structure. Therefore, the recrystallization effect and preferential melting of small grains are not sufficient to form fine spherical grains. In fact, the heterogeneous nucleation and Zener pinning effect of second-phase particles also played a critical role in the formation of spherical grains. Figure 7 presents the spherical grains’ formation process. The static recrystallization [15] occurred firstly in the heated microstructures accompanied by the non-dendritic equiaxed grains, whereas, following low melting point alloy phases, the grain boundaries and sharp corners of the solid phases were melted to form the liquid phases. These liquid phases appeared isolated from each other in the non-dendritic equiaxed grain boundaries and grain inclusions, whereas, consequently, a higher number of liquid phases appeared. All liquid phases migrated to the neighboring films as the temperature increased. When the temperature reaches the set temperature for holding sometimes, the liquid phase migration was followed by heterogeneous nucleation with temperature dropping [16,17].

## 5. Conclusions

The formation mechanism of spherical grains and orientation evolution in semi-solid state of C5191 copper alloy were studied by in situ observation of deformation-induced spherical grains. The main conclusions could be obtained as follows:

1. HT-CLSM was used to in situ observe the rotary swaged material and raw material without deformation at the same heating cycle. Compared with the microstructural evolution of the two materials, the formation process of spherical grains of semi-solid material includes the following stages: static recrystallization occurred firstly to form equiaxed recrystallized grains, whereas following the intersection of grain boundaries and small grains melted priority. These liquid phases appeared isolated from each other in the equiaxed grain boundaries and grain inclusions at the early stage, and then liquid phase migration. When the temperature reaches the semi-solid state, spherical grains are formed. Finally, heterogeneous nucleation solidification and Zener pining hinder the free growth of spherical grains with temperature dropping.
2. The mechanism formation of spherical grains is a synergetic mechanism of grain refinement and grain spheroidization, including static recrystallization, preferential melting of small grains and grain boundaries, heterogeneous nucleation in solidification, and the Zener pinning effect of second-phase particles during the SIMA process.

## References

[1] Vieira E.A., Kliauga A.M., Ferrante M. (2007). On the formation of spheroidal microstructures in a semi-solid Al–Si alloy by thermomechanical processing. Scr. Mater..

[2] Natori K., Utsunomiya H., Tanaka T. (2017). Improvement in formability of semi-solid cast hypoeutectic Al-Si alloys by equal-channel angular pressing. J. Mater. Process. Technol..

[3] Boroujeny B.S., Ghashghaei M., Akbari E. (2018). Effects of SIMA (Strain Induced Melt Activation) on microstructure and electrochemical behavior of Al-Zn-In sacrificial anodes. J. Alloys Compd..

[4] Atkinson H.V., Burke K., Vaneetveld G. (2008). Recrystallisation in the semi-solid state in 7075 aluminium alloy. Mater. Sci. Eng. A.

[5] Gu G., Pesci R., Langlois L., Becker E., Bigot R., Guo M. (2014). Microstructure observation and quantification of the liquid fraction of M2 steel grade in the semi-solid state, combining confocal laser scanning microscopy and X-ray microtomography. Acta Mater..

[6] Lin H., Wang J.G., Wang H., Jiang Q. (2007). Effect of predeformation on the globular grains in AZ91D alloy during strain induced melt activation (SIMA) process. J. Alloys Compd..

[7] Hassas-Irani S., Zarei-Hanzaki A., Bazaz B., Roostaei A.A. (2013). Microstructure evolution and semi-solid deformation behavior of an A356 aluminum alloy processed by strain induced melt activated method. Mater. Des..

[8] Sirong Y., Dongcheng L., Kim N. (2006). Microstructure evolution of SIMA processed Al2024. Mater. Sci. Eng. A.

[9] Abdulstaar M., El-Danaf E.A., Waluyo N.S., Wagner L. (2013). Severe plastic deformation of commercial purity aluminum by rotary swaging: Microstructure evolution and mechanical properties. Mater. Sci. Eng. A.

[10] Cao M., Wang Z., Zhang Q. (2017). Microstructure-dependent mechanical properties of semi-solid copper alloys. J. Alloys Compd..

[11] Clark S., Janik V., Rijkenberg A., Sridhar S. (2016). Analysis of the extent of interphase precipitation in V-HSLA steels through in-situ characterization of the γ/α transformation. Mater. Charact..

[12] Attallah M.M., Terasaki H., Moat R., Bray S., Komizo Y., Preuss M. (2011). In-Situ observation of primary γ′ melting in Ni-base superalloy using confocal laser scanning microscopy. Mater. Charact..

[13] Huang K., Logé R. (2016). A review of dynamic recrystallization phenomena in metallic materials. Mater. Des..

[14] Cao M., Zhang Q., Huang K., Wang X., Chang B., Cai L. (2020). Microstructural evolution and deformation behavior of copper alloy during rheoforging process. J. Mater. Sci. Technol..

[15] Moelans N., Blanpain B., Wollants P. (2006). Phase field simulations of grain growth in two-dimensional systems containing finely dispersed second-phase particles. Acta Mater..

[16] Cai Y., Tan M., Shen G., Su H. (2000). Microstructure and heterogeneous nucleation phenomena in cast SiC particles reinforced magnesium composite. Mater. Sci. Eng. A.

[17] Humphreys F., Hatherly M. (2004). Recrystallization and Related Annealing Phenomena.

[18] Huang K., Marthinsen K., Zhao Q.-L., Logé R.E. (2018). The double-edge effect of second-phase particles on the recrystallization behaviour and associated mechanical properties of metallic materials. Prog. Mater. Sci..

[19] Rios P.R., Jr F.S., Sandim H., Plaut R.L., Padilha A.F. (2005). Nucleation and growth during recrystallization. Mater. Res..

[20] Paul H., Driver J., Jasieński Z. (2002). Shear banding and recrystallization nucleation in a Cu–2%Al alloy single crystal. Acta Mater..

[21] Cao M., Zhang Q., Zhang Y. (2017). Effects of plastic energy on thixotropic microstructure of C5191 alloys during SIMA process. J. Alloys Compd..

[22] Kurz W., Fisher D. (1998). Fundamentals of Solidification.

[23] Murty B.S., Kori S.A., Chakraborty M. (2002). Grain refinement of aluminium and its alloys by heterogeneous nucleation and alloying. Int. Mater. Rev..

[24] Fan Z., Lin X., Xu R., Dong Y., Wen B., Wang L., Zhao S. (2018). Electron back-scattering diffraction preliminary analysis of heterogeneous nuclei in magnesium alloy during solidification process under GPa high pressure. J. Rare Earths.

